# Modulating Oral Microbiota to Prevent Dental Caries: A Microbial Ecology Approach

**DOI:** 10.3390/dj14080477

**Published:** 2026-08-04

**Authors:** Yu-Chen Lee, Yu-Che Cheng, Chun-Ming Kung, Chi-Jung Huang

**Affiliations:** 1 Division of Family Dentistry, Oral Medicine Department, Cathay General Hospital, Taipei 106438, Taiwan; cgh12673@cgh.org.tw; 2 Department of Medical Research, Cathay General Hospital, Taipei 106438, Taiwan; yccheng@cgh.org.tw; 3 Department of Biomedical Science and Engineering, National Central University, Taoyuan 320317, Taiwan; 4 Division of Oral Implantology, Oral Medicine Department, Cathay General Hospital, Taipei 106438, Taiwan; 5 Department and Graduate Institute of Biochemistry, College of Biomedical Sciences, National Defense Medical University, Taipei 114201, Taiwan

**Keywords:** oral microbiota, caries prevention, acidogenic bacteria, probiotics and prebiotics, microbiome diagnostics

## Abstract

**Background**: Dental caries is a highly prevalent, biofilm-mediated disease characterized by microbial dysbiosis, excessive acid production, and progressive enamel demineralization. Although traditionally managed through restorative treatment, increasing attention has shifted toward preventive strategies focused on modulation of the oral microbiota and maintenance of ecological balance within the oral cavity. **Methods**: This narrative review summarizes current evidence regarding the ecological and mechanistic basis of dental caries and microbiota-centered prevention strategies. Literature published between January 2000 and March 2026 was retrieved from PubMed/MEDLINE, Scopus, Web of Science, and Google Scholar using keywords related to dental caries, oral microbiota, cariogenic bacteria, biofilms, probiotics, prebiotics, salivary diagnostics, metabolomics, quorum sensing, and artificial intelligence. **Results**: Current evidence demonstrates that dental caries is driven by ecological shifts favoring acidogenic and aciduric microorganisms within cariogenic biofilms. Emerging preventive approaches include dietary modification, oral hygiene optimization, probiotics, prebiotics, synbiotics, and functional dietary agents aimed at restoring microbial homeostasis and inhibiting cariogenic biofilm maturation. In addition, advances in salivary microbiome profiling, metabolomics, artificial intelligence-assisted predictive modeling, and smart responsive materials have shown promising potential for improving early diagnosis, risk assessment, and personalized prevention strategies. **Conclusions**: Microbiota-based approaches represent a promising paradigm shift in dental caries prevention by emphasizing ecological modulation rather than pathogen eradication alone. Continued interdisciplinary research integrating microbial ecology, diagnostics, biomaterials, and digital technologies may facilitate the development of personalized and preventive oral healthcare strategies.

## 1. Introduction

Dental caries remains one of the most prevalent chronic conditions worldwide, affecting individuals across all age groups [1]. Once viewed primarily as a consequence of poor oral hygiene and high sugar consumption, caries is now understood as a complex, biofilm-mediated disease rooted in microbial dysbiosis [2]. The transition from health to disease involves a shift in the ecological balance of the dental plaque biofilm, wherein acidogenic and aciduric species dominate, producing localized low-pH environments conducive to enamel demineralization [3].

At the center of this dysbiosis is the microbial metabolism of fermentable carbohydrates, most notably sucrose, into organic acids such as lactic acid [4]. These acids contribute to a persistent acidic microenvironment, favoring the proliferation of acid-tolerant species like *Streptococcus mutans*, *Lactobacillus* spp., and *Scardovia wiggsiae* [3,5,6]. High-throughput microbial sequencing shows caries is polymicrobial, supporting the ecological plaque hypothesis that disease results from an imbalance in the microbial community rather than a single pathogen [7,8].

**Figure 1:**
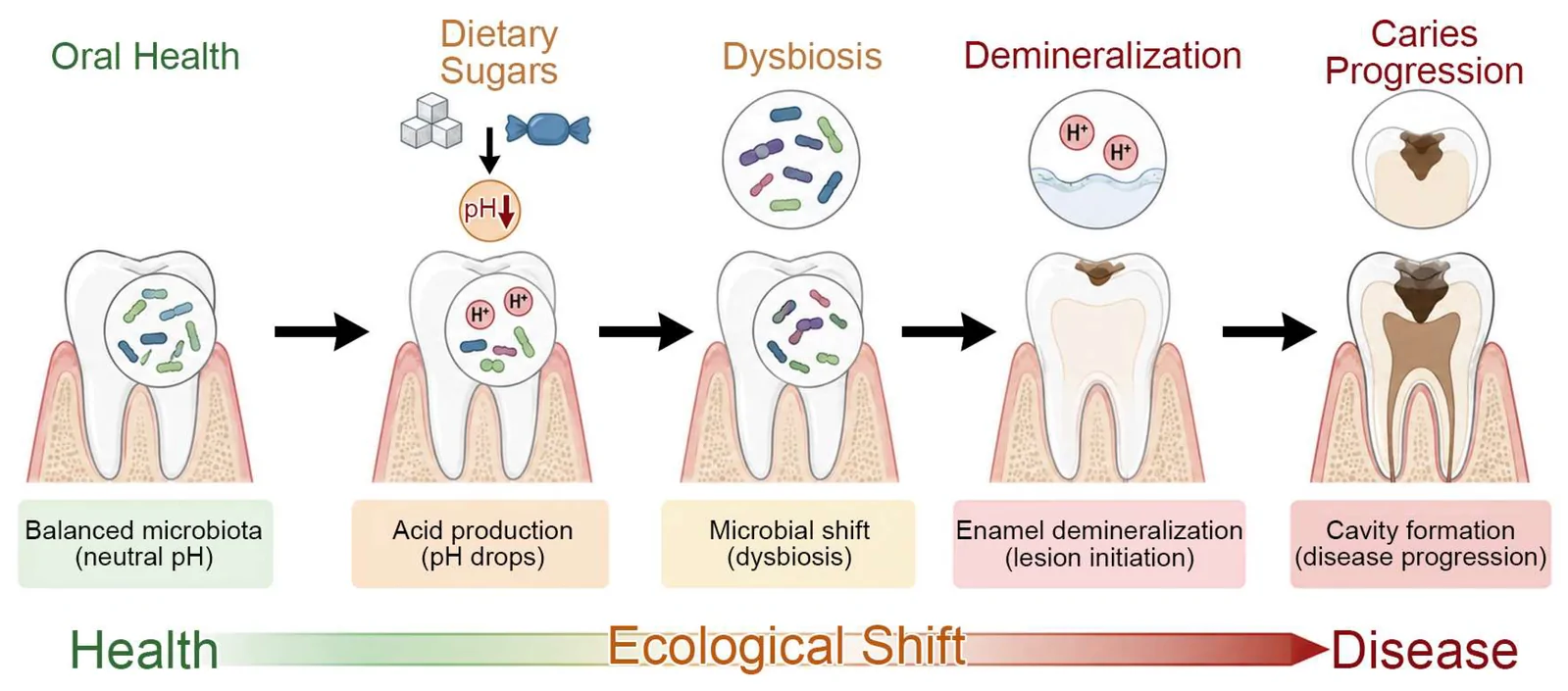
Schematic overview of the ecological shift from oral microbial homeostasis to cariogenic dysbiosis and dental caries following frequent dietary sugar exposure.

Given this framework, prevention should aim to restore and maintain microbial homeostasis rather than merely eliminate pathogens [2,9]. Strategies include promoting alkali-generating commensals, limiting substrates for acidogenic organisms, and enhancing biofilm resilience through non-cariogenic pathways [10,11]. This review is organized into four interconnected themes. First, we summarize the ecological mechanisms underlying cariogenic dysbiosis and biofilm development. We then discuss microbiota-based preventive strategies aimed at restoring oral ecological balance, followed by emerging diagnostic technologies for caries risk assessment. Finally, we highlight future perspectives in personalized microbiota-driven prevention. Throughout this review, we emphasize that effective caries prevention is achieved not through eradication of individual pathogens but through preservation and restoration of oral microbial homeostasis. The ecological transition from oral health to cariogenic dysbiosis, characterized by shifts in microbial composition and localized acidification, is illustrated in Figure 1.

## 2. Materials and Methods

### 2.1. Review Design

This study was conducted as a narrative review aimed at summarizing current evidence regarding the role of oral microbiota modulation in the prevention of dental caries. The review focused on ecological mechanisms of cariogenesis, microbiota-based preventive strategies, and emerging diagnostic technologies relevant to oral microbial dysbiosis.

### 2.2. Literature Search Strategy

A comprehensive literature search was performed using the electronic databases PubMed/MEDLINE, Scopus, Web of Science, and Google Scholar. Articles published in English between January 2000 and March 2026 were considered. The search strategy combined Medical Subject Headings (MeSH) terms and free-text keywords, including: “dental caries,” “oral microbiota,” “oral microbiome,” “cariogenic bacteria,” “biofilm,” “*Streptococcus mutans*,” “acidogenic bacteria,” “aciduric bacteria,” “probiotics,” “prebiotics,” “synbiotics,” “salivary microbiome,” “metabolomics,” “quorum sensing,” and “artificial intelligence.” Boolean operators (“AND” and “OR”) were used to optimize the search. Reference lists of relevant articles were additionally screened to identify supplementary studies.

### 2.3. Eligibility Criteria

Articles were included if they investigated mechanisms associated with cariogenic microbial dysbiosis, examined microbiota-targeted preventive or therapeutic approaches for dental caries, evaluated diagnostic or predictive tools related to the oral microbiome, or provided clinically and biologically relevant insights into oral microbial ecology and caries prevention. Original research articles, clinical studies, systematic reviews, meta-analyses, and significant experimental studies were considered eligible for inclusion. Non-English publications, conference abstracts without available full texts, duplicate records, and studies not directly related to oral microbiota or caries prevention were excluded from the review.

### 2.4. Data Extraction and Synthesis

Relevant information from eligible studies was extracted and organized according to thematic categories, including cariogenic microbiota, ecological shifts, biofilm formation, microbiota-based preventive interventions, salivary diagnostics, metabolomics, and artificial intelligence-assisted predictive models. The findings were narratively synthesized to provide an integrated overview of current evidence and emerging perspectives in microbiota-centered caries prevention.

## 3. Cariogenic Microbiota and Ecological Shifts

Dental caries is now recognized as a polymicrobial ecological disease resulting from dysbiosis within the oral biofilm rather than the activity of a single pathogen. Although *Streptococcus mutans* remains one of the best-characterized cariogenic species because of its acidogenicity and aciduricity, it acts within a complex microbial community that includes other acidogenic and acid-tolerant bacteria contributing to biofilm maturation and disease progression.

### 3.1. Cariogenic Microbiota

Dental caries is a polymicrobial ecological disease characterized by dysbiosis within the oral biofilm. Among the best-characterized cariogenic bacteria, *Streptococcus mutans* plays a major role through its acidogenicity, aciduricity, and production of exopolysaccharides (EPSs) [12,13]. However, cariogenicity arises from coordinated interactions among multiple microbial species that collectively promote biofilm maturation and acidification [14]. These bacteria possess mechanisms that not only enable them to thrive in acidic environments created by the fermentation of dietary sugars but also facilitate their adhesion to dental surfaces, contributing to biofilm formation [12]. For instance, *Streptococcus mutans* synthesizes glucan-type EPSs, which enhance the biofilm’s stability and cohesion, creating a habitat where other cariogenic species can proliferate [15]. The biochemical nature of these biofilms, primarily reflected in their EPS composition, significantly influences their cariogenic potential. The major cariogenic bacterial species and their pathogenic characteristics are summarized in Table 1. Studies have illustrated that biofilms rich in insoluble glucans demonstrate a higher propensity to lower the pH in the dental environment, which is critical for enamel demineralization [16].

Moreover, the ecological dynamics of cariogenic microbiota highlight its polymicrobial nature, where species such as *Lactobacillus* spp. and *Actinomyces* also contribute to caries progression by enhancing acidogenic potential [20]. The interplay among these species, including *Rothia* sp., indicates a complex biofilm community that exhibits traits associated with cariogenicity, including acid tolerance and EPS production [7,12]. Such interactions between various bacteria underscore the necessity for a multifaceted approach in understanding cariogenic mechanisms and developing therapeutic strategies. For example, treatments targeting the biofilm’s microenvironment to regulate pH levels and inhibit EPS formation have emerged as promising areas for future research [10,24]. Through these multifarious interactions and metabolic events, cariogenic microbiota poses significant challenges to oral health, necessitating continued exploration for effective preventative and therapeutic interventions.

### 3.2. Acidogenic and Aciduric Adaptation

Cariogenic microbiota, particularly in the context of dental caries, exhibit adaptations that enhance their acidogenicity and aciduricity [3,25]. Acidogenic bacteria, most notably mutans streptococci such as *Streptococcus mutans*, are capable of metabolizing sugars to produce organic acids that lower the pH of the oral environment, promoting the demineralization of dental enamel [26,27]. This process is critical in initiating carious lesions, as frequent access to sugars can drive the ecological shift towards a biofilm dominated by acidogenic and aciduric species [22,28]. Aciduric species, which can survive and thrive in acidic conditions, reflect an adaptive response to the carious environment, indicating a balance alteration within the biofilm community [29]. For instance, non-mutans streptococci and lactobacilli also contribute to acid production and have been shown to increase in proportion following episodes of sugar consumption [14,30]. Research has demonstrated that bacterial adaptation to low pH involves complex physiological changes. For instance, *Streptococcus oralis* exhibits adaptive responses under acidic conditions that support survival and metabolic activity within cariogenic biofilms [31,32]. Furthermore, studies indicate that acid-adapted bacterial strains exhibit higher rates of growth and acid production when re-exposed to acidic environments, reinforcing the idea that the dental biofilm can become progressively more aciduric in response to dietary habits and local pH fluctuations [33]. The dynamics of these microbial communities illustrate that mixed species interactions can optimize their collective acidogenic potential, thereby maintaining a detrimental environment conducive to caries development [19]. Over time, the continuous acidification from these bacteria results in selection pressure favoring more aciduric organisms, ultimately exacerbating the severity of carious lesions [34]. The interplay between acidogenicity and aciduricity within cariogenic microbiota significantly influences the caries process, characterized by a gradual shift towards more acid-tolerant species that enhance tooth demineralization. As these bacteria adapt to the acidic milieu, they not only survive but thrive, furthering their cariogenic potential and perpetuating the cycle of dental decay [21].

## 4. Mechanisms Driving Caries Progression

Cariogenicity arises from coordinated interactions among multiple microbial species through metabolic cross-feeding, extracellular matrix formation, quorum sensing, and ecological adaptation, rather than from the activity of a single organism [14].

### 4.1. EPS Production and Biofilm Architecture

The progression of dental caries is closely linked to the architecture of biofilms formed by cariogenic microbes, particularly *Streptococcus mutans* [15]. EPSs are produced in high quantities during the metabolism of fermentable carbohydrates, particularly sucrose [35]. The resulting biofilm architecture allows for the formation of dense, structured communities that enhance bacterial adhesion to tooth surfaces and facilitate the retention of nutrients, further promoting an environment conducive to cariogenic activity [36]. The mechanisms driving caries progression through EPS production are multifaceted. Enhanced EPS production not only provides structural integrity to the biofilm but also creates localized acidic microenvironments due to the accumulation of metabolic byproducts from acidogenic bacteria [37,38]. The presence of sucrose leads to the synthesis of insoluble glucans, which serve as a scaffold for the biofilm, allowing *Streptococcus mutans* and other acidogenic species to flourish [39]. This architectural predominance of *Streptococcus mutans* in biofilms enables the establishment of microcolonies that can thrive in acidic conditions, underscoring their aciduricity, a crucial characteristic for survival in the cariogenic milieu [18]. Moreover, the structural dynamics of these biofilms affect diffusion properties and the availability of essential nutrients, which can inhibit the growth of beneficial bacteria, further tipping the ecological balance toward pathogenic species [40]. As carbohydrate exposure continues, the resulting acidic conditions, partly facilitated by the biofilm architecture, encourage the persistence of aciduric organisms, contributing to the overall virulence of the biofilm and accelerating the demineralization of dental hard tissues [41].

### 4.2. Suppression of Alkali-Producing Species

Dental caries develops more rapidly when alkali-producing bacterial species in the oral microbiome are suppressed [42]. Typically, the advancement of caries is associated with an increase in acidogenic bacteria, which thrive in low-pH environments and contribute to the demineralization of tooth structures [29]. Recent studies highlight that a balanced oral microbiome, where alkali-producing species such as *Streptococcus sanguinis* and other commensal bacteria are prevalent, is crucial for maintaining pH homeostasis and inhibiting caries onset [42,43]. For instance, alkali production from salivary substrates, particularly arginine and urea, serves a protective role by neutralizing the acids generated by cariogenic bacteria through carbohydrate metabolism [42]. This ability to produce ammonia and other basic metabolites directly opposes the acidic milieu fostered by pathogens such as *Streptococcus mutans* [29,44]. When the population of alkali-producing bacteria declines, the oral environment shifts towards one dominated by aciduric organisms, which exacerbates the acidic conditions and triggers further mineral loss. The data collected by Du et al. illustrate that high sucrose concentrations can preferentially reduce alkali-generating bacteria, thereby disrupting the delicate balance between acid and base producers, thus accelerating caries development [45]. Furthermore, alkali production from biofilm communities has been shown to enhance bioenergetic responses and offer protection against acid-induced damage, which is critical for maintaining dental health [23]. Collectively, these findings suggest that enhancing the presence and metabolic activity of alkali-producing bacterial species could represent a viable strategy for caries management and prevention [46].

### 4.3. Quorum Sensing and Biofilm Resilience

The progression of dental caries is critically influenced by various mechanisms, among which quorum sensing (QS) and biofilm resilience are paramount [47]. QS facilitates communication among *Streptococcus mutans* populations, allowing them to coordinate virulence factor production and biofilm formation in response to the density of their bacterial community [48]. Specifically, the Competing Stimulating Peptide-mediated QS system in *Streptococcus mutans* regulates multiple virulence-associated traits, such as the production of bacteriocins intended to eliminate competing species and enhance the pathogen’s dominance in dental biofilms [49]. This communal behavior leads to the establishment of robust biofilms that are resistant to external stressors, including antimicrobial agents, as conventional treatments often fail to disrupt these resilient structures effectively [50]. The resilient nature of dental biofilms is attributed to the structural complexity of these microbial communities, which provide a protective environment for *Streptococcus mutans*, allowing it to thrive in acidic conditions that occur from carbohydrate fermentation [51]. Moreover, the interaction of *Streptococcus mutans* with other species, including commensal bacteria, contributes to biofilm stability and alters its susceptibility to dental treatments [52]. Strategies aimed at disrupting QS signals, such as quorum quenching with plant-derived compounds, have been proposed as novel therapeutic avenues to counteract *Streptococcus mutans* biofilm formation and mitigate the persistent threat of dental caries [53].

### 4.4. Synergistic Interactions and Acid Tolerance

**Figure 2:**
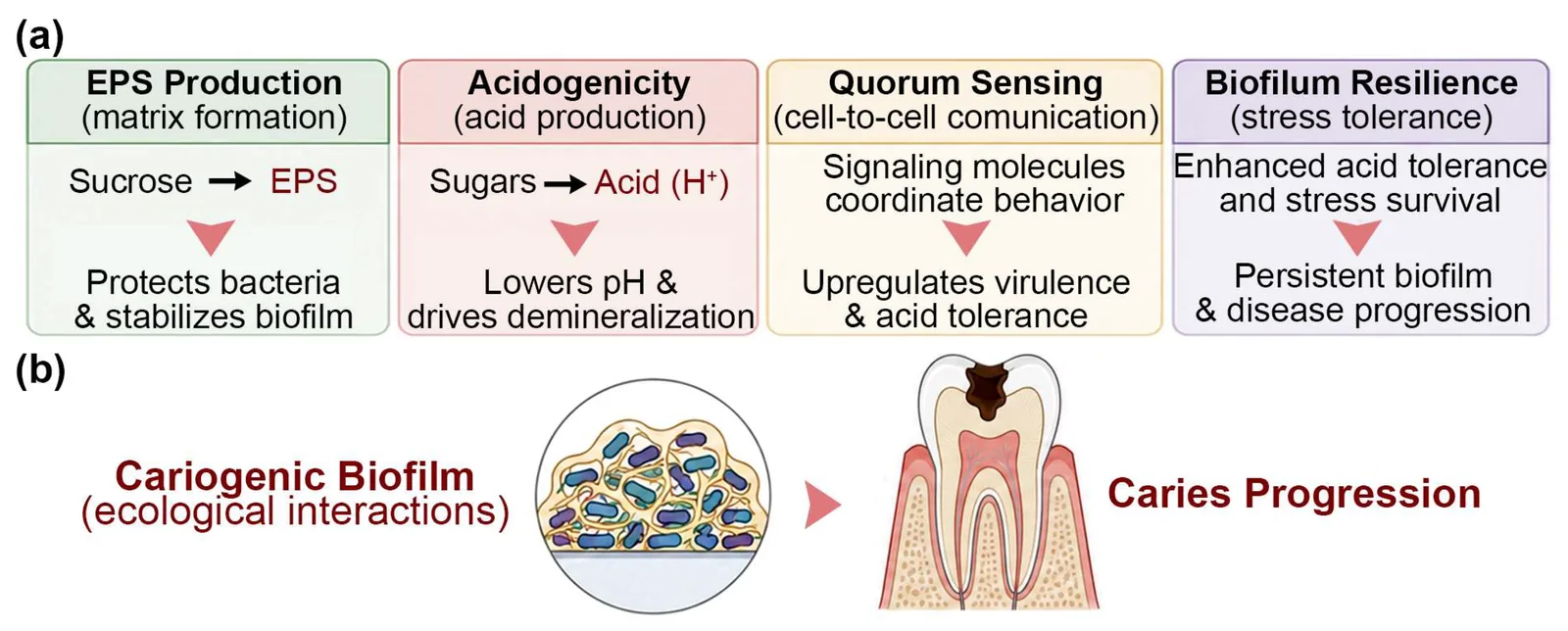
Mechanisms underlying cariogenic biofilm development. (**a**) Major mechanisms promoting cariogenic biofilm formation, including EPS production, acidogenicity, quorum sensing, and biofilm resilience. (**b**) Their synergistic interactions drive caries progression. EPS, exopolysaccharide.

The development of dental caries is largely driven by how biofilm microbes interact and their acid tolerance, both of which affect oral ecological balance [29]. The “Ecological Plaque Hypothesis” posits that shifts in microbial composition, driven by dietary changes such as increased consumption of fermentable carbohydrates, lead to the proliferation of acidogenic bacteria [54]. Within this framework, the intake of sugars like sucrose not only promotes the growth of cariogenic pathogens, such as *Streptococcus mutans*, but also disrupts the microecological equilibrium that typically supports non-pathogenic species, thereby enhancing dysbiosis [55]. This dysbiosis is characterized by an abundance of acidogenic bacteria, which thrive in low-pH conditions, exacerbating the acidic microenvironment necessary for dental demineralization [56]. Importantly, acid tolerance mechanisms among cariogenic bacteria reinforce their survival and pathogenic potential. For instance, the persistence of *Streptococcus mutans* in acidic environments allows for sustained biofilm formation and acid production, which, in turn, promotes further selection of aciduric bacteria [57]. As biofilms age, the biofilm composition often shifts towards aciduric species due to a selective advantage conferred by their acid tolerance, thus perpetuating an environment conducive to carious activity [58]. This ecological advantage is essential; the glucan-rich matrix produced by *Streptococcus mutans* not only aids in bacterial cohesion but also creates localized acidic microenvironments that are detrimental to tooth enamel [17]. Thus, understanding the synergistic interactions between acidogenic bacteria and the mechanisms of acid tolerance is crucial for developing effective strategies aimed at mitigating or reversing caries progression. The interconnected roles of EPS production, acidogenicity, QS, and biofilm resilience in caries progression are summarized in Figure 2. Figure 2a illustrates the key virulence-associated mechanisms, including EPS production, acidogenicity, QS, and biofilm resilience, whereas Figure 2b depicts the synergistic interactions within cariogenic biofilms leading to enamel destruction and progressive dental caries.

## 5. Microbiota-Based Prevention Strategies

**Figure 3:**
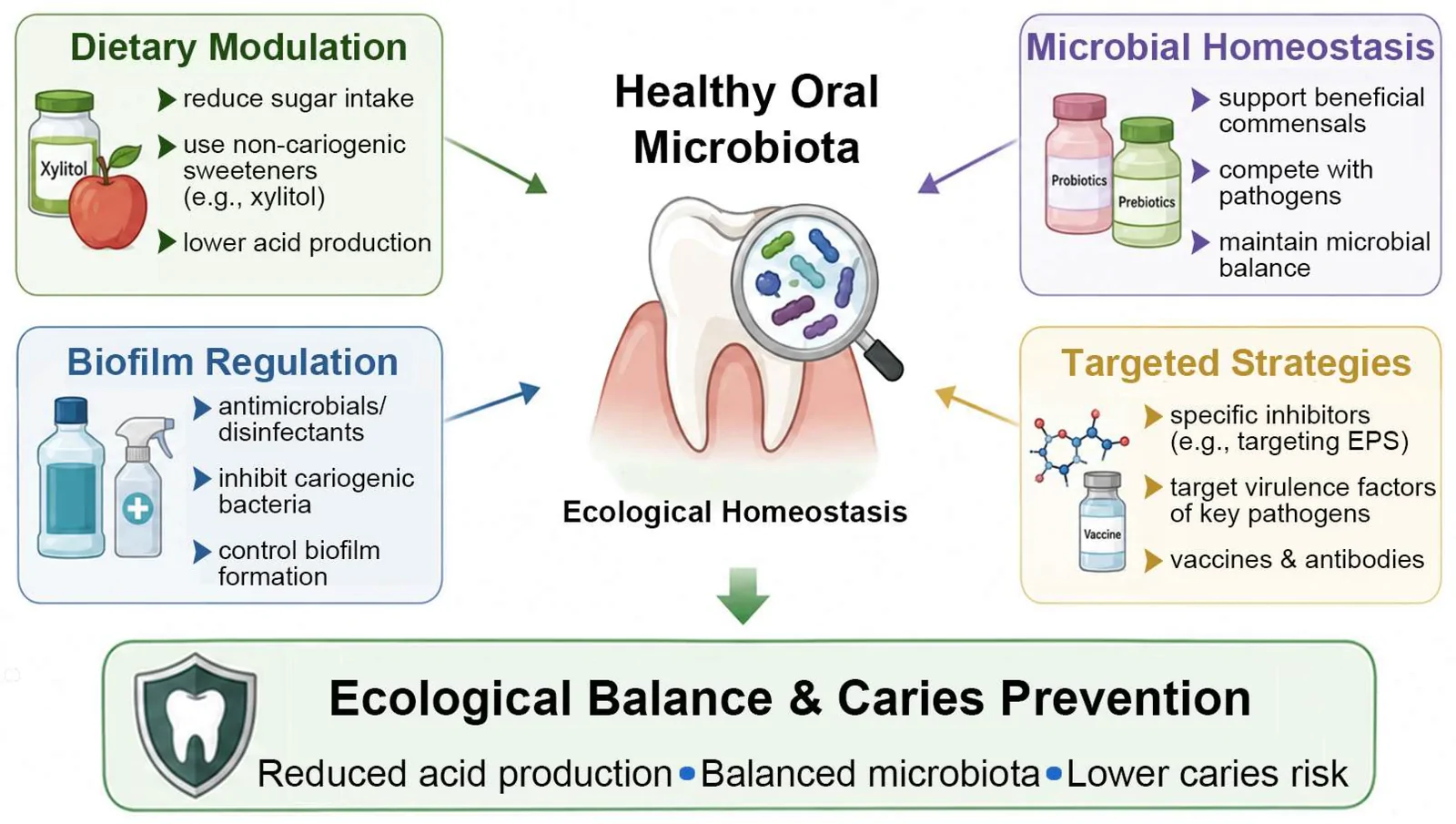
Microbiota-centered strategies for maintaining oral ecological homeostasis and preventing dental caries. Dietary modulation, biofilm regulation, microbial homeostasis, and targeted anti-cariogenic approaches collectively promote a balanced oral microbiota and reduce caries risk. EPS, exopolysaccharide.

Figure 3 summarizes microbiota-centered preventive strategies aimed at maintaining ecological balance through dietary modulation, biofilm regulation, and preservation of microbial homeostasis. Current microbiota-based preventive interventions and their proposed mechanisms of action are further outlined in Table 2.

### 5.1. Dietary Modulation

#### 5.1.1. Reducing Fermentable Carbohydrates

Microbiota-based prevention strategies in dental health increasingly emphasize the reduction in fermentable carbohydrates, which play a significant role in dental caries development [10,80]. The metabolism of these carbohydrates by cariogenic bacteria, such as *Streptococcus mutans*, leads to the production of organic acids, creating an acidic microenvironment that facilitates tooth enamel demineralization [72]. To effectively mitigate this risk, a comprehensive approach that includes microbiota modulation is essential. Probiotics have emerged as a potential adjunctive strategy, where specific strains can outcompete harmful bacteria for available nutrients and space, thereby inhibiting their pathogenicity [70]. Probiotics and synbiotics can enhance oral health by promoting a balanced microbiome capable of resisting acidogenic challenges brought about by high sugar intake, thus reducing the likelihood of caries formation [68]. Additionally, integrating dietary interventions to limit the intake of fermentable carbohydrates, particularly sugars, is critical to these microbiota-centered prevention methodologies [29]. The synergistic use of probiotics alongside dietary strategies may yield better outcomes in caries prevention by not only reducing substrates available for pathogenic bacteria but also enhancing the resilience of health-promoting microbial species within the oral cavity [81]. Various studies underscore the need for further research to establish the effectiveness of probiotic therapy paired with dietary modifications to ensure comprehensive prevention strategies targeting the complex relationship between carbohydrates and oral microbial health [73].

#### 5.1.2. Functional Dietary Agents

Strategies that use microbiota for dental health have become increasingly popular, with an emphasis on functional dietary substances like xylitol, arginine, and polyphenols to support oral health and help prevent cavities [10,82]. Xylitol, a five-carbon sugar alcohol, is recognized for its ability to reduce the levels of *Streptococcus mutans*, a key bacterium associated with dental caries [60]. Research has demonstrated that xylitol not only inhibits the growth of cariogenic bacteria but also promotes saliva flow, which is critical for enamel remineralization, thereby playing a protective role against caries development [60]. Furthermore, it has been proposed that xylitol functions as an oral prebiotic by fostering a beneficial oral microbiota composition [55]. Arginine, on the other hand, is noted for its prebiotic properties, contributing to the alkalinization of the oral environment, which may discourage acidogenic bacterium growth [43]. Its effectiveness has been corroborated through studies indicating that arginine-modified dental products can lead to favorable changes in the oral microbiome, enhancing the presence of beneficial bacteria while suppressing pathogenic species [61]. These modifications are particularly pertinent for individuals susceptible to caries, showing how targeted dietary interventions can ameliorate dysbiosis and promote oral health [59]. Polyphenols, derived mainly from plant sources, have shown antimicrobial properties that can inhibit the growth of harmful bacteria while simultaneously promoting the growth of beneficial microorganisms [63]. Their role as modulators of gut and oral microbiota suggests potential for therapeutic applications in managing dysbiosis and enhancing the microbiota’s resilience, directly impacting oral health outcomes [64].

### 5.2. Oral Hygiene Practices

Microbiota-based prevention strategies in dental health emphasize the importance of maintaining a balanced oral microbiota through effective oral hygiene practices, particularly tooth brushing [9,83]. The oral microbiota plays a crucial role in oral and systemic health, with its dysbiosis contributing to various oral diseases such as dental caries and periodontal disease [84]. Regular and effective tooth brushing disrupts pathogenic biofilm formation on teeth, which is essential for preventing these conditions [85]. Research highlights that inadequate oral hygiene can alter the oral microbiota, resulting in increased caries risk due to the proliferation of cariogenic bacteria [86]. The mechanical removal of plaque through brushing is considered the “gold standard” for preventing both caries and periodontal diseases, as it effectively manages biofilm accumulation and maintains a healthier oral environment [87]. Furthermore, interventions addressing oral hygiene education, particularly in children, have proven effective in favorably altering microbiota profiles, thereby decreasing the prevalence of dental caries. This multifaceted approach is bolstered by evidence suggesting that enhancing the presence of beneficial bacteria and managing diet through education can further improve oral health outcomes [88]. Therefore, promoting proper oral hygiene practices, especially tooth brushing, is integral in managing the oral microbiota and preventing dental diseases.

### 5.3. Probiotics and Prebiotics

#### 5.3.1. Probiotics

Microbiota-based prevention strategies, particularly through the use of probiotics, have shown promising potential in the maintenance of oral health and the prevention of dental diseases, including caries and periodontal issues [89]. Research indicates that certain probiotic strains, such as *Lactobacillus* and *Bifidobacterium* species, can effectively compete with cariogenic bacteria like *Streptococcus mutans*, thereby reducing their population and potential for cavity formation [68]. Moreover, the efficacy of probiotic interventions can extend beyond mere competition; probiotics may also improve the overall health of the oral cavity by modulating the immune response and producing antimicrobial compounds, which further inhibit pathogen growth [71].

Studies have demonstrated that consumption of probiotics, for instance in the form of fermented dairy products or supplements, can support salivary flow and promote enamel remineralization, which are critical factors in reducing the risk of dental caries [67,69]. Additionally, the synergistic use of synbiotics, which combine probiotics and prebiotics, has been highlighted as a viable strategy for enhancing the stability of beneficial oral microflora while compromising pathogenic bacteria [90]. However, it is important to note that the clinical application of probiotics in dental care remains in its developmental stages, with studies often limited to short-term efficacy and lacking long-term follow-up. There exists a potential challenge in achieving consistent colonization in the oral cavity due to the complexity of the established oral microbiota; thus, ongoing research is aimed at identifying specific probiotic strains that can effectively integrate into the oral ecosystem and confer lasting benefits [74]. The eventual goal is to develop personalized oral health strategies that utilize specific probiotic formulations tailored to individual microbiome profiles, thereby enhancing the potential for disease prevention in dental health management [66].

#### 5.3.2. Prebiotics

Prebiotics, defined as non-digestible food ingredients that selectively foster beneficial microorganisms, play a crucial role in promoting a balanced and healthy oral microbiota, which is essential in preventing dental diseases like caries and periodontitis [91]. For example, certain prebiotics such as arginine and urea have shown potential in generating alkaline conditions conducive to reducing acid-producing pathogenic bacteria in the mouth, thus tilting the microbial balance towards health-associated species [62]. Studies suggest that prebiotics like nitrate can effectively modulate the oral microbiome’s composition by decreasing the prevalence of periodontopathic bacteria, thereby contributing to oral health maintenance [68]. Moreover, the synergistic application of prebiotics along with probiotics enhances their efficacy, as prebiotics can improve the growth and activity of probiotics, resulting in a more significant impact on the oral ecosystem [92]. As research continues to illustrate the intricate relationship between the oral microbiota and health, implementing prebiotic strategies within holistic dental care practices is gaining recognition for potentially transforming traditional views on preventing and managing oral diseases [93].

#### 5.3.3. Synbiotics

The efficacy of synbiotics in promoting a healthy oral microbiome aids in the management of existing conditions and plays a preventive role by restoring balance to microbial populations disrupted by poor dietary habits or antibiotic use [94]. Furthermore, synbiotics have demonstrated potential in enhancing the immune response within the oral cavity, promoting the growth of beneficial bacterial strains that can inhibit pathogen colonization and reduce inflammation [95]. This dual action, both prevention and management, underlines the importance of integrating synbiotics into traditional dental care practices. The exploration of novel formulations, such as those containing *Lactobacillus* and *Bifidobacterium* species, exemplifies the potential of synbiotics to offer innovative therapeutic solutions to enhance oral health and prevent systemic diseases linked to oral microbiota dysbiosis [65,73].

### 5.4. Current Evidence and Clinical Considerations

Although microbiota-based preventive strategies have shown considerable promise, the strength of supporting evidence varies substantially among different interventions. Dietary modification, reduction in fermentable carbohydrate intake, fluoride therapy, and xylitol are supported by relatively strong clinical evidence, including randomized controlled trials and systematic reviews, and are currently incorporated into preventive dental practice. In contrast, probiotics, prebiotics, and synbiotics have demonstrated encouraging biological effects in vitro and in animal models, with several clinical studies reporting reductions in *Streptococcus mutans* levels or modest improvements in caries-related outcomes. However, the overall clinical evidence remains heterogeneous because of differences in probiotic strains, formulations, treatment durations, study populations, and outcome measures. Consequently, current systematic reviews generally conclude that these microbiota-targeted interventions are promising adjunctive approaches but that high-quality, long-term randomized clinical trials are still needed before routine clinical implementation can be broadly recommended.

Similarly, functional dietary agents such as arginine and plant-derived polyphenols exhibit favorable ecological effects by promoting alkali production, suppressing virulence factors, or modulating biofilm composition. Nevertheless, much of the supporting evidence remains preclinical, and additional well-designed clinical studies are required to establish their long-term efficacy and optimal clinical application. Overall, contemporary evidence supports a shift from broad-spectrum antimicrobial approaches toward ecological biofilm modulation. Rather than indiscriminately eliminating oral microorganisms, microbiota-based interventions aim to selectively suppress cariogenic activities while preserving beneficial microbial communities and restoring oral ecological homeostasis. Consequently, these strategies should be regarded as complementary approaches that enhance, rather than replace, established preventive measures [96].

## 6. Diagnostic and Predictive Tools

Emerging diagnostic and predictive approaches for caries risk assessment are summarized in Table 3.

### 6.1. Salivary Microbiome Profiling

The salivary microbiome has emerged as an important diagnostic and predictive tool in assessing dental caries, particularly due to its noninvasive nature and the rich information it provides about oral health status [114]. Recent studies highlight distinct microbial profiles in saliva associated with dental caries, which could serve as valuable biomarkers for diagnosing and predicting caries progression [29]. For instance, Vieira et al. demonstrated that children with active dental caries exhibited a unique salivary microbiome composition when compared to their caries-free counterparts, reinforcing the potential of salivary profiles in diagnosing caries status [101]. Similarly, Xu et al. reported longitudinal shifts in the oral microbiome of young children transitioning from caries-free to caries-affected, suggesting that changes in microbiota could precede clinical manifestations of dental caries [103]. Furthermore, the interplay between salivary metabolites and microbial diversity has shown promise, with Kim et al. identifying specific metabolic changes linked to caries that may lead to the discovery of novel salivary biomarkers for early detection [99]. The presence of certain bacterial taxa, such as *Streptococcus mutans* and *Prevotella*, has been shown to correlate with caries status, potentially enabling targeted interventions based on individual salivary microbiome profiles [97,100]. The integration of advanced analytical techniques, like mass spectrometry, enhances the capacity to explore the salivary proteome, offering insights into protein biomarkers that could aid in personalized monitoring of dental caries [98,102]. Collectively, these developments indicate that salivary microbiome profiling may revolutionize the diagnostic landscape for dental caries, facilitating earlier infection detection and more tailored treatment approaches. Despite its considerable diagnostic potential, routine clinical application of salivary microbiome profiling remains limited by variability in sample collection, sequencing methodologies, and bioinformatic analysis. Further standardization and multicenter clinical validation are required before it can be routinely incorporated into personalized caries-risk assessment.

### 6.2. Metabolomic and pH Biomarkers

Recent advancements in metabolomic analyses, particularly through nuclear magnetic resonance and mass spectrometry, have facilitated the identification of potential biomarkers in saliva that correlate with caries status, showcasing the potential for novel diagnostic avenues in dental health management [115,116]. Studies indicate that salivary metabolite profiles, influenced by microbial dysbiosis, can reflect the state of oral health and predict the risk of developing dental caries [107]. For example, specific metabolites have been associated with shifts in the local microbiome, revealing a complex interplay between salivary biochemistry and microbial presence [99]. Moreover, pH levels serve as critical indicators of dental caries activity, with lower salivary pH consistently linked to higher caries prevalence. Research shows that individuals with active dental caries often exhibit notably acidic pH ranges, between 5.0 and 5.8, which are detrimental to enamel health and promote microbial proliferation [23,105]. The role of salivary flow rate and buffering capacity in maintaining optimal pH levels also emerges in the literature as a vital factor in caries susceptibility, providing insight into the mechanistic aspects of salivary influence on oral health [106]. Thus, integrating pH monitoring with metabolic profiling offers a comprehensive approach for early detection and intervention strategies in managing dental caries, underscoring the need for further exploration within this rapidly evolving research domain [104]. However, routine clinical implementation remains limited by high cost, technical complexity, and the lack of standardized analytical protocols. Large-scale clinical validation will be essential before metabolomic biomarkers can be widely adopted in dental practice.

### 6.3. AI and Predictive Modeling

The application of AI in the diagnosis and prediction of dental caries is rapidly gaining momentum, with numerous studies highlighting its potential impact on oral health management [113,117]. AI techniques, particularly deep learning algorithms, have demonstrated accuracy in analyzing dental radiographs, thereby facilitating earlier and more precise detection of carious lesions [118,119]. For example, convolutional neural networks have been reported to achieve accuracy rates of up to 97% with low false positive rates in identifying carious lesions, enhancing diagnostic reliability [108,120]. Such advancements could significantly augment traditional diagnostic methods, thereby improving preventive strategies for conditions like pulpitis and abscess formation that arise from poorly diagnosed caries [110]. Moreover, the speed of AI diagnostic processes often surpasses that of experienced clinicians, allowing for quicker evaluations of radiographic images, which could contribute to improved patient throughput and diagnostic efficiency in dental practices [109]. Additionally, predictive modeling frameworks utilizing extensive datasets are being explored to forecast future caries development, emphasizing the importance of data quality in training these models to ensure reliable outcomes [111]. Although AI has considerable potential to improve personalized caries prediction and diagnostic efficiency, its clinical implementation requires further external validation, standardized datasets, and careful consideration of data privacy, reproducibility, and regulatory issues. At present, AI should be regarded as a complementary tool rather than a replacement for clinical judgment [112].

## 7. Future Perspectives

### 7.1. Personalized Microbiota-Driven Prevention

The development of dental caries is influenced by complex interactions among pathogenic microorganisms, dietary behaviors, and host-related factors [2]. Research shows that changes in tongue microbiota relate to oral health, directly linking microbiome composition with dental caries prevalence [121]. Furthermore, personalized preventive strategies may incorporate dietary modifications and probiotics targeting specific dysbiotic species to restore ecological balance within the oral cavity [122]. Advances in proteomics reveal that salivary proteins interact with oral microbiota, affecting caries risk and offering potential biomarkers for assessment [123]. Understanding the dynamics of oral biofilms and their ecological relationships allows for tailoring interventions that can mitigate the risks associated with dysbiosis [75]. Thus, a microbiota-driven approach toward caries prevention emphasizes not only the reduction in pathogenic organisms but also the promotion of beneficial microbiotic communities as a pathway to enhance oral health.

### 7.2. Smart and Responsive Materials

Dental caries, a common oral disease marked by tooth decay resulting from demineralization caused by acid-producing bacteria, is being increasingly managed with the advancement of smart and responsive materials [124]. These materials are designed to be antibacterial and react to changes in oral pH. For example, the incorporation of smart dental materials that react to local acidic conditions can help inhibit the growth of cariogenic bacteria, thereby reducing the incidence of caries [79]. The ability of these materials to release bioactive ions or antimicrobial agents in response to acidic pH enhances their efficacy in remineralizing enamel and dentin, providing a dual benefit of antibacterial action and mineral restoration [125]. Advancements in this field include the development of innovative coatings and composite materials designed to inhibit biofilm formation, a key factor in caries progression, through mechanisms that either repel bacterial adhesion or exert bactericidal effects upon contact [126]. Furthermore, the integration of nanotechnology into smart dental materials, as seen in the development of metallic and polymeric nanocomposites, has shown promise in improving antibacterial efficacy through multifaceted action [77,78]. These materials not only enhance treatment outcomes but may also lead to a shift toward more preventive and less invasive treatment modalities, aligning with contemporary trends in minimally invasive dentistry [76]. Despite these promising developments, most smart materials remain at the experimental or early clinical stage. Further studies evaluating long-term performance, cost-effectiveness, regulatory approval, and chairside applicability are needed before widespread clinical adoption.

## 8. Conclusions

Modulating the oral microbiota represents a biologically grounded and sustainable strategy for preventing dental caries. By leveraging advances in microbial ecology, diagnostics, and materials science, dentistry is transitioning from reactive restoration to proactive prevention. Although microbiota-based preventive and diagnostic strategies have demonstrated considerable promise, their levels of clinical evidence and readiness for implementation vary substantially. Continued efforts toward methodological standardization, multicenter clinical validation, cost-effectiveness evaluation, and regulatory approval will be essential before these emerging technologies can be widely integrated into personalized caries prevention. Continued interdisciplinary research will be vital in realizing the full potential of personalized, microbiota-centered care.

## Figures and Tables

**Table 1:** Key cariogenic bacteria and their roles.

Bacterial Species	Characteristics	Pathogenic Role	References
*Streptococcus mutans*	Acidogenic, aciduric, produces EPSs ^1^	Initiates caries, forms robust biofilm structure	[15,17,18]
*Lactobacillus* spp.	Highly acidogenic and aciduric	Associated with lesion progression, especially in dentin	[19,20]
*Scardovia wiggsiae*	Emerging pathogen, strong acid tolerance	Linked to early childhood caries	[19,21]
*Bifidobacterium dentium*	Produces lactate and acetate	Implicated in root caries	[15,22]
*Veillonella* spp.	Utilizes lactic acid from others	Supports acid production via metabolic cross-feeding	[15,23]

^1^ EPSs, exopolysaccharides.

**Table 2:** Preventive agents and their mechanisms.

Agent/Intervention	Category	Mechanism of Action	Current Evidence	Major Limitation	References
Xylitol	Dietary polyol	Inhibits *Streptococcus mutans* adhesion, reduces acid production	Moderate–strong	Compliance dependent	[55,59,60]
Arginine	Prebiotic	Promotes alkali production, stabilizes plaque pH	Moderate	Limited long-term RCTs ^3^	[43,44,61,62]
Polyphenols (e.g., tea, cranberry)	Dietary phytochemicals	Inhibit EPS synthesis, downregulate virulence factors ^1^	Mainly/preclinical	Lack of standardized formulations	[56,63,64]
Fluoride	Topical agent	Enhances remineralization, suppresses acidogenicity	Strong	Does not directly restore microbial ecology	[46,56,63,64,65]
Probiotics (*L. rhamnosus*, *S. salivarius*)	Microbiota-based	Reduce *Streptococcus mutans* colonization, shift microbiota composition	Moderate/heterogeneous results	Strain-specific/inconsistent colonization	[66,67,68,69,70,71]
Synbiotics	Microbiota-based	Combined probiotic–prebiotic effects	Emerging	Few large multicenter trials	[68,72,73]
CPC ^2^	Antiseptic	Targets *Streptococcus mutans* while preserving commensals	Moderate	Possible short-term microbiota perturbation	[65,74]
Enzyme-based/nano-silver gels	Emerging hygiene	Selectively inhibit cariogenic species without disrupting microbiota	Experimental/ preclinical	Cost and limited clinical evidence	[75,76,77,78,79]

^1^ EPS, exopolysaccharide. ^2^ CPC, cetylpyridinium chloride. ^3^ RCTs, randomized controlled trials.

**Table 3:** Diagnostic tools for caries risk assessment.

Tool/Method	Principle	Clinical Utility	References
Salivary microbiome profiling	NGS to identify microbial Composition ^1^	Detects at-risk microbiota patterns	[97,98,99,100,101,102,103]
pH sensing technologies	Real-time biofilm acidity monitoring	Assesses acidogenic potential after sugar exposure	[23,104,105,106]
VOC analysis ^2^ (e.g., lactic acid)	Measures metabolic by-products	Indicates active cariogenic activity	[99,107]
Salivary buffering tests	Quantifies neutralizing capacity	Evaluates host defense against acid challenges	[104,106]
AI-based predictive Modeling ^3^	Combines biological and behavioral data	Enables personalized prevention and population screening	[108,109,110,111,112,113]
Microfluidic/colorimetric devices	Portable diagnostic platforms	Supports chairside or public-health use	[111,112]

^1^ NGS, next-generation sequencing. ^2^ VOC, volatile organic compound. ^3^ AI, artificial intelligence.

## Data Availability

The original contributions presented in this study are included in the article. Further inquiries can be directed to the corresponding authors.

## Abbreviations

The following abbreviations are used in this manuscript:

EPS	exopolysaccharide
QS	quorum sensing

## References

[1] Peres M.A., Macpherson L.M.D., Weyant R.J., Daly B., Venturelli R., Mathur M.R., Listl S., Celeste R.K., Guarnizo-Herreno C.C., Kearns C. (2019). Oral diseases: A global public health challenge. Lancet.

[2] Pitts N.B., Zero D.T., Marsh P.D., Ekstrand K., Weintraub J.A., Ramos-Gomez F., Tagami J., Twetman S., Tsakos G., Ismail A. (2017). Dental caries. Nat. Rev. Dis. Prim..

[3] Takahashi N., Nyvad B. (2011). The role of bacteria in the caries process: Ecological perspectives. J. Dent. Res..

[4] Paes Leme A.F., Koo H., Bellato C.M., Bedi G., Cury J.A. (2006). The role of sucrose in cariogenic dental biofilm formation—New insight. J. Dent. Res..

[5] Marsh P.D. (2003). Are dental diseases examples of ecological catastrophes?. Microbiology.

[6] Tanner A.C., Mathney J.M., Kent R.L., Chalmers N.I., Hughes C.V., Loo C.Y., Pradhan N., Kanasi E., Hwang J., Dahlan M.A. (2011). Cultivable anaerobic microbiota of severe early childhood caries. J. Clin. Microbiol..

[7] Gross E.L., Beall C.J., Kutsch S.R., Firestone N.D., Leys E.J., Griffen A.L. (2012). Beyond *Streptococcus mutans*: Dental caries onset linked to multiple species by 16S rRNA community analysis. PLoS ONE.

[8] Simon-Soro A., Mira A. (2015). Solving the etiology of dental caries. Trends Microbiol..

[9] Marsh P.D. (2012). Contemporary perspective on plaque control. Br. Dent. J..

[10] Philip N., Suneja B., Walsh L.J. (2018). Ecological Approaches to Dental Caries Prevention: Paradigm Shift or Shibboleth?. Caries Res..

[11] Marsh P.D. (2018). In Sickness and in Health-What Does the Oral Microbiome Mean to Us? An Ecological Perspective. Adv. Dent. Res..

[12] Bowen W.H., Koo H. (2011). Biology of *Streptococcus mutans*-derived glucosyltransferases: Role in extracellular matrix formation of cariogenic biofilms. Caries Res..

[13] Krzysciak W., Jurczak A., Koscielniak D., Bystrowska B., Skalniak A. (2014). The virulence of *Streptococcus mutans* and the ability to form biofilms. Eur. J. Clin. Microbiol. Infect. Dis..

[14] Spatafora G., Li Y., He X., Cowan A., Tanner A.C.R. (2024). The Evolving Microbiome of Dental Caries. Microorganisms.

[15] Wang Y., Wang S., Wu C., Chen X., Duan Z., Xu Q., Jiang W., Xu L., Wang T., Su L. (2019). Oral Microbiome Alterations Associated with Early Childhood Caries Highlight the Importance of Carbohydrate Metabolic Activities. mSystems.

[16] Toro E., Nascimento M.M., Suarez-Perez E., Burne R.A., Elias-Boneta A., Morou-Bermudez E. (2010). The effect of sucrose on plaque and saliva urease levels in vivo. Arch. Oral Biol..

[17] Klein M.I., Hwang G., Santos P.H., Campanella O.H., Koo H. (2015). *Streptococcus mutans*-derived extracellular matrix in cariogenic oral biofilms. Front. Cell. Infect. Microbiol..

[18] Lemos J.A., Palmer S.R., Zeng L., Wen Z.T., Kajfasz J.K., Freires I.A., Abranches J., Brady L.J. (2019). The Biology of *Streptococcus mutans*. Microbiol. Spectr..

[19] Hurley E., Barrett M.P.J., Kinirons M., Whelton H., Ryan C.A., Stanton C., Harris H.M.B., O’Toole P.W. (2019). Comparison of the salivary and dentinal microbiome of children with severe-early childhood caries to the salivary microbiome of caries-free children. BMC Oral Health.

[20] Mei M.L., Li Q.L., Chu C.H. (2016). Inhibition of Cariogenic Plaque Formation on Root Surface with Polydopamine-Induced-Polyethylene Glycol Coating. Materials.

[21] Escobar-Arregocés F., Eras M.A., Bustos A., Suárez-Castillo A., García-Robayo D.A., Del Pilar Bernal M. (2024). Characterization of the oral microbiota and the relationship of the oral microbiota with the dental and periodontal status in children and adolescents with nonsyndromic cleft lip and palate. Systematic literature review and meta-analysis. Clin. Oral Investig..

[22] Takahashi N., Nyvad B. (2016). Ecological Hypothesis of Dentin and Root Caries. Caries Res..

[23] Edlund A., Yang Y., Yooseph S., Hall A.P., Nguyen D.D., Dorrestein P.C., Nelson K.E., He X., Lux R., Shi W. (2015). Meta-omics uncover temporal regulation of pathways across oral microbiome genera during in vitro sugar metabolism. ISME J..

[24] Koo H., Falsetta M.L., Klein M.I. (2013). The exopolysaccharide matrix: A virulence determinant of cariogenic biofilm. J. Dent. Res..

[25] Liu Y., Ren Z., Hwang G., Koo H. (2018). Therapeutic Strategies Targeting Cariogenic Biofilm Microenvironment. Adv. Dent. Res..

[26] Head D., Devine A.D., Marsh P.D. (2017). In silico modelling to differentiate the contribution of sugar frequency versus total amount in driving biofilm dysbiosis in dental caries. Sci. Rep..

[27] Korithoski B., Lévesque C.M., Cvitkovitch D.G. (2008). The involvement of the pyruvate dehydrogenase E1alpha subunit, in *Streptococcus mutans* acid tolerance. FEMS Microbiol. Lett..

[28] Hassan H., Lingström P., Carlén A. (2015). Plaque pH in caries-free and caries-active young individuals before and after frequent rinses with sucrose and urea solution. Caries Res..

[29] Liu R., Liu Y., Yi J., Fang Y., Guo Q., Cheng L., He J., Li M. (2025). Imbalance of oral microbiome homeostasis: The relationship between microbiota and the occurrence of dental caries. BMC Microbiol..

[30] Wen Z.T., Huang X., Ellepola K., Liao S., Li Y. (2022). Lactobacilli and human dental caries: More than mechanical retention. Microbiology.

[31] Boisen G., Davies J.R., Neilands J. (2021). Acid tolerance in early colonizers of oral biofilms. BMC Microbiol..

[32] Okahashi N., Nakata M., Kuwata H., Kawabata S. (2022). Oral mitis group streptococci: A silent majority in our oral cavity. Microbiol. Immunol..

[33] Senneby A., Davies J.R., Svensäter G., Neilands J. (2017). Acid tolerance properties of dental biofilms in vivo. BMC Microbiol..

[34] Liu Y.L., Nascimento M., Burne R.A. (2012). Progress toward understanding the contribution of alkali generation in dental biofilms to inhibition of dental caries. Int. J. Oral Sci..

[35] Livne S., Simantov S., Rahmanov A., Jeffet U., Sterer N. (2022). Hydroxyethyl Cellulose Promotes the Mucin Retention of Herbal Extracts Active against *Streptococcus mutans*. Materials.

[36] Falsetta M.L., Klein M.I., Lemos J.A., Silva B.B., Agidi S., Scott-Anne K.K., Koo H. (2012). Novel antibiofilm chemotherapy targets exopolysaccharide synthesis and stress tolerance in *Streptococcus mutans* to modulate virulence expression in vivo. Antimicrob. Agents Chemother..

[37] Hwang G., Liu Y., Kim D., Sun V., Aviles-Reyes A., Kajfasz J.K., Lemos J.A., Koo H. (2016). Simultaneous spatiotemporal mapping of in situ pH and bacterial activity within an intact 3D microcolony structure. Sci. Rep..

[38] Sun Y., Pan Y., Sun Y., Li M., Huang S., Qiu W., Tu H., Zhang K. (2019). Effects of Norspermidine on Dual-Species Biofilms Composed of *Streptococcus mutans* and *Streptococcus sanguinis*. BioMed Res. Int..

[39] Philip N., Bandara H., Leishman S.J., Walsh L.J. (2019). Inhibitory effects of fruit berry extracts on *Streptococcus mutans* biofilms. Eur. J. Oral Sci..

[40] Duarte S., Klein M.I., Aires C.P., Cury J.A., Bowen W.H., Koo H. (2008). Influences of starch and sucrose on *Streptococcus mutans* biofilms. Oral Microbiol. Immunol..

[41] Florez Salamanca E.J., Klein M.I. (2018). Extracellular matrix influence in *Streptococcus mutans* gene expression in a cariogenic biofilm. Mol. Oral Microbiol..

[42] Del Rey Y.C., Rikvold P.D., Lund M.B., Raittio E.J., Schramm A., Meyer R.L., Schlafer S. (2025). Arginine modulates the pH, microbial composition, and matrix architecture of biofilms from caries-active patients. Int. J. Oral Sci..

[43] Nascimento M.M. (2018). Potential Uses of Arginine in Dentistry. Adv. Dent. Res..

[44] Burne R.A., Marquis R.E. (2000). Alkali production by oral bacteria and protection against dental caries. FEMS Microbiol. Lett..

[45] Du Q., Fu M., Zhou Y., Cao Y., Guo T., Zhou Z., Li M., Peng X., Zheng X., Li Y. (2020). Sucrose promotes caries progression by disrupting the microecological balance in oral biofilms: An in vitro study. Sci. Rep..

[46] Ferrazzano G.F., Cantile T., Ingenito A., Chianese L., Quarto M. (2007). New strategies in dental caries prevention: Experimental study on casein phosphopeptides. Eur. J. Paediatr. Dent..

[47] Wright P.P., Ramachandra S.S. (2022). Quorum Sensing and Quorum Quenching with a Focus on Cariogenic and Periodontopathic Oral Biofilms. Microorganisms.

[48] Fitri D.K., Tuygunov N., Wan Harun W.H.A., Purwasena I.A., Cahyanto A., Zakaria M.N. (2025). Key virulence genes associated with *Streptococcus mutans* biofilm formation: A systematic review. Front. Oral Health.

[49] Wyllie R.M., Jensen P.A. (2025). The MutRS quorum-sensing system controls lantibiotic mutacin production in the human pathogen *Streptococcus mutans*. Proc. Natl. Acad. Sci. USA.

[50] Nijampatnam B., Ahirwar P., Pukkanasut P., Womack H., Casals L., Zhang H., Cai X., Michalek S.M., Wu H., Velu S.E. (2021). Discovery of Potent Inhibitors of *Streptococcus mutans* Biofilm with Antivirulence Activity. ACS Med. Chem. Lett..

[51] Li Y.H., Hanna M.N., Svensäter G., Ellen R.P., Cvitkovitch D.G. (2001). Cell density modulates acid adaptation in *Streptococcus mutans*: Implications for survival in biofilms. J. Bacteriol..

[52] Matsumoto-Nakano M. (2018). Role of *Streptococcus mutans* surface proteins for biofilm formation. Jpn. Dent. Sci. Rev..

[53] Paluch E., Rewak-Soroczyńska J., Jędrusik I., Mazurkiewicz E., Jermakow K. (2020). Prevention of biofilm formation by quorum quenching. Appl. Microbiol. Biotechnol..

[54] Marsh P.D., Zaura E. (2017). Dental biofilm: Ecological interactions in health and disease. J. Clin. Periodontol..

[55] Zhan L. (2018). Rebalancing the Caries Microbiome Dysbiosis: Targeted Treatment and Sugar Alcohols. Adv. Dent. Res..

[56] Vijayakumar A., Sarveswari H.B., Vasudevan S., Shanmugam K., Solomon A.P., Neelakantan P. (2021). Baicalein Inhibits *Streptococcus mutans* Biofilms and Dental Caries-Related Virulence Phenotypes. Antibiotics.

[57] Oliveira S.G., Jardim R., Kotowski N., Dávila A.M.R., Sampaio-Filho H.R., Ruiz K.G.S., Aguiar F.H.B. (2025). The Dentin Microbiome: A Metatranscriptomic Evaluation of Caries-Associated Bacteria. Biomedicines.

[58] Boisen G., Brogårdh-Roth S., Neilands J., Mira A., Carda-Diéguez M., Davies J.R. (2024). Oral biofilm composition and phenotype in caries-active and caries-free children. Front. Oral Health.

[59] Loimaranta V., Mazurel D., Deng D., Söderling E. (2020). Xylitol and erythritol inhibit real-time biofilm formation of *Streptococcus mutans*. BMC Microbiol..

[60] Söderling E.M. (2009). Xylitol, mutans streptococci, and dental plaque. Adv. Dent. Res..

[61] Ghesquière J., Simoens K., Koos E., Boon N., Teughels W., Bernaerts K. (2023). Spatiotemporal monitoring of a periodontal multispecies biofilm model: Demonstration of prebiotic treatment responses. Appl. Environ. Microbiol..

[62] Zaura E., Twetman S. (2019). Critical Appraisal of Oral Pre- and Probiotics for Caries Prevention and Care. Caries Res..

[63] Correia M., Gomes A., Moreira I., El Maghariki J., Mendes K., Correia M.J., Barros R., Barbosa J.C., Rosa N., Gomes A.M. (2025). Unraveling the Extra Virgin Olive Oil Effect on Inflammation and on Gut and Saliva Microbiota. Biomolecules.

[64] Molinari R., Merendino N., Costantini L. (2022). Polyphenols as modulators of pre-established gut microbiota dysbiosis: State-of-the-art. Biofactors.

[65] Yu O.Y., Lam W.Y., Wong A.W., Duangthip D., Chu C.H. (2021). Nonrestorative Management of Dental Caries. Dent. J..

[66] Homayouni Rad A., Pourjafar H., Mirzakhani E. (2023). A comprehensive review of the application of probiotics and postbiotics in oral health. Front. Cell. Infect. Microbiol..

[67] Hu X., Huang Z., Zhang Y., Hong Y., Zheng Y. (2019). Effects of a probiotic drink containing *Lactobacillus casei* strain Shirota on dental plaque microbiota. J. Int. Med. Res..

[68] Luo S.C., Wei S.M., Luo X.T., Yang Q.Q., Wong K.H., Cheung P.C.K., Zhang B.B. (2024). How probiotics, prebiotics, synbiotics, and postbiotics prevent dental caries: An oral microbiota perspective. npj Biofilms Microbiomes.

[69] Romani Vestman N., Chen T., Lif Holgerson P., Öhman C., Johansson I. (2015). Oral Microbiota Shift after 12-Week Supplementation with *Lactobacillus reuteri* DSM 17938 and PTA 5289; A Randomized Control Trial. PLoS ONE.

[70] Seminario-Amez M., López-López J., Estrugo-Devesa A., Ayuso-Montero R., Jané-Salas E. (2017). Probiotics and oral health: A systematic review. Med. Oral Patol. Oral Cir. Bucal.

[71] Xu J., Chen C., Gan S., Liao Y., Fu R., Hou C., Yang S., Zheng Z., Chen W. (2023). The Potential Value of Probiotics after Dental Implant Placement. Microorganisms.

[72] Bijle M.N., Ekambaram M., Lo E.C.M., Yiu C.K.Y. (2020). Synbiotics in caries prevention: A scoping review. PLoS ONE.

[73] Twetman S., Belstrøm D. (2025). Effect of Synbiotic and Postbiotic Supplements on Dental Caries and Periodontal Diseases-A Comprehensive Review. Int. J. Environ. Res. Public Health.

[74] Hoare A., Marsh P.D., Diaz P.I. (2017). Ecological Therapeutic Opportunities for Oral Diseases. Microbiol. Spectr..

[75] de Sousa F.F., Ferraz C., Rodrigues L.K., Nojosa Jde S., Yamauti M. (2014). Nanotechnology in dentistry: Drug delivery systems for the control of biofilm-dependent oral diseases. Curr. Drug Deliv..

[76] Montoya C., Roldan L., Yu M., Valliani S., Ta C., Yang M., Orrego S. (2023). Smart dental materials for antimicrobial applications. Bioact. Mater..

[77] Su Y., Wang J., Wang Y., Han X., Liu Y., Gong H., Zhang M., Ma S., Zhao J. (2025). Zeolitic Imidazolate Framework-8 as a pH-Responsive Smart Targeted Release Nanoplatform for Antibacterial Therapy and Wound Regeneration. Chem. Asian J..

[78] Wu Y., Liu P., Mehrjou B., Chu P.K. (2024). Interdisciplinary-Inspired Smart Antibacterial Materials and Their Biomedical Applications. Adv. Mater..

[79] Yu K., Zhang Q., Dai Z., Zhu M., Xiao L., Zhao Z., Bai Y., Zhang K. (2023). Smart Dental Materials Intelligently Responding to Oral pH to Combat Caries: A Literature Review. Polymers.

[80] Moynihan P., Tanner L.M., Holmes R.D., Hillier-Brown F., Mashayekhi A., Kelly S.A.M., Craig D. (2019). Systematic Review of Evidence Pertaining to Factors That Modify Risk of Early Childhood Caries. JDR Clin. Trans. Res..

[81] Lopes P.C., Gomes A., Mendes K., Blanco L., Correia M.J. (2024). Unlocking the potential of probiotic administration in caries management: A systematic review. BMC Oral Health.

[82] Mira A., Simon-Soro A., Curtis M.A. (2017). Role of microbial communities in the pathogenesis of periodontal diseases and caries. J. Clin. Periodontol..

[83] Rosier B.T., Marsh P.D., Mira A. (2018). Resilience of the Oral Microbiota in Health: Mechanisms That Prevent Dysbiosis. J. Dent. Res..

[84] Khan I.M., Mani S.A., Doss J.G., Danaee M., Kong L.Y.L. (2021). Pre-schoolers’ tooth brushing behaviour and association with their oral health: A cross sectional study. BMC Oral Health.

[85] Patel C., Patel M.C., Joshi S.S., Mehta M., Makwani D., Patel F., Bale J., Solanki C. (2025). Impact of Sleep and Associated Factors on the Prevalence of Early Childhood Caries: An Analytical Cross-Sectional Study. Cureus.

[86] Xu H., Tian B., Shi W., Tian J., Wang W., Qin M. (2022). Maturation of the oral microbiota during primary teeth eruption: A longitudinal, preliminary study. J. Oral Microbiol..

[87] Arweiler N.B., Netuschil L. (2016). The Oral Microbiota. Adv. Exp. Med. Biol..

[88] Muhoozi G.K.M., Li K., Atukunda P., Skaare A.B., Willumsen T., Enersen M., Westerberg A.C., Morris A., Vieira A.R., Iversen P.O. (2022). Child saliva microbiota and caries: A randomized controlled maternal education trial in rural Uganda. Sci. Rep..

[89] Inchingolo F., Inchingolo A.M., Malcangi G., De Leonardis N., Sardano R., Pezzolla C., de Ruvo E., Di Venere D., Palermo A., Inchingolo A.D. (2023). The Benefits of Probiotics on Oral Health: Systematic Review of the Literature. Pharmaceuticals.

[90] Rodriguez G.A., Cabello R.A., Borroni C.P., Palacio R.A. (2022). Cost-effectiveness of probiotics and fluoride varnish in caries prevention in preschool children. J. Public Health Dent..

[91] Yadav M.K., Kumari I., Singh B., Sharma K.K., Tiwari S.K. (2022). Probiotics, prebiotics and synbiotics: Safe options for next-generation therapeutics. Appl. Microbiol. Biotechnol..

[92] Turska-Szybka A., Olczak-Kowalczyk D., Twetman S. (2025). Probiotics, Prebiotics, Synbiotics, and Postbiotics Against Oral Candida in Children: A Review of Clinical Trials. Nutrients.

[93] Rajasekaran J.J., Krishnamurthy H.K., Bosco J., Jayaraman V., Krishna K., Wang T., Bei K. (2024). Oral Microbiome: A Review of Its Impact on Oral and Systemic Health. Microorganisms.

[94] Bijle M.N., Neelakantan P., Ekambaram M., Lo E.C.M., Yiu C.K.Y. (2020). Effect of a novel synbiotic on *Streptococcus mutans*. Sci. Rep..

[95] Wei X., Qian S., Yang Y., Mo J. (2024). Microbiome-based therapies for periodontitis and peri-implantitis. Oral Dis..

[96] D’Agostino S., Valentini G., Iarussi F., Dolci M. (2024). Effect of Probiotics *Lactobacillus rhamnosus* and *Lactobacillus plantarum* on Caries and Periodontal Diseases: A Systematic Review. Dent. J..

[97] Jiang S., Gao X., Jin L., Lo E.C. (2016). Salivary Microbiome Diversity in Caries-Free and Caries-Affected Children. Int. J. Mol. Sci..

[98] Khan Z.M., Waheed H., Khurshid Z., Zafar M.S., Moin S.F., Alam M.K. (2021). Differentially Expressed Salivary Proteins in Dental Caries Patients. BioMed Res. Int..

[99] Kim S., Song Y., Kim S., Kim S., Na H., Lee S., Chung J., Kim S. (2023). Identification of a Biomarker Panel for Diagnosis of Early Childhood Caries Using Salivary Metabolic Profile. Metabolites.

[100] Lee E., Park S., Um S., Kim S., Lee J., Jang J., Jeong H.O., Shin J., Kang J., Lee S. (2021). Microbiome of Saliva and Plaque in Children According to Age and Dental Caries Experience. Diagnostics.

[101] Vieira A.R., Hiller N.L., Powell E., Kim L.H., Spirk T., Modesto A., Kreft R. (2019). Profiling microorganisms in whole saliva of children with and without dental caries. Clin. Exp. Dent. Res..

[102] Vukosavljevic D., Custodio W., Siqueira W.L. (2011). Salivary proteins as predictors and controls for oral health. J. Cell Commun. Signal.

[103] Xu H., Tian J., Hao W., Zhang Q., Zhou Q., Shi W., Qin M., He X., Chen F. (2018). Oral Microbiome Shifts From Caries-Free to Caries-Affected Status in 3-Year-Old Chinese Children: A Longitudinal Study. Front. Microbiol..

[104] Animireddy D., Reddy Bekkem V.T., Vallala P., Kotha S.B., Ankireddy S., Mohammad N. (2014). Evaluation of pH, buffering capacity, viscosity and flow rate levels of saliva in caries-free, minimal caries and nursing caries children: An in vivo study. Contemp. Clin. Dent..

[105] Lin X., Wang Y., Ma Z., Xie M., Liu Z., Cheng J., Tian Y., Shi H. (2023). Correlation between caries activity and salivary microbiota in preschool children. Front. Cell. Infect. Microbiol..

[106] Lin Y.J., Lin Y.T. (2018). Influence of dental plaque pH on caries status and salivary microflora in children following comprehensive dental care under general anesthesia. J. Dent. Sci..

[107] Gardner A., Carpenter G., So P.W. (2020). Salivary Metabolomics: From Diagnostic Biomarker Discovery to Investigating Biological Function. Metabolites.

[108] Albano D., Galiano V., Basile M., Di Luca F., Gitto S., Messina C., Cagetti M.G., Del Fabbro M., Tartaglia G.M., Sconfienza L.M. (2024). Artificial intelligence for radiographic imaging detection of caries lesions: A systematic review. BMC Oral Health.

[109] Bağ İ., Bilgir E., Bayrakdar İ., Baydar O., Atak F.M., Çelik Ö., Orhan K. (2023). An artificial intelligence study: Automatic description of anatomic landmarks on panoramic radiographs in the pediatric population. BMC Oral Health.

[110] Das M., Shahnawaz K., Raghavendra K., Kavitha R., Nagareddy B., Murugesan S. (2024). Evaluating the Accuracy of AI-Based Software vs Human Interpretation in the Diagnosis of Dental Caries Using Intraoral Radiographs: An RCT. J. Pharm. Bioallied Sci..

[111] Kishimoto T., Goto T., Matsuda T., Iwawaki Y., Ichikawa T. (2022). Application of artificial intelligence in the dental field: A literature review. J. Prosthodont. Res..

[112] Rischke R., Schneider L., Müller K., Samek W., Schwendicke F., Krois J. (2022). Federated Learning in Dentistry: Chances and Challenges. J. Dent. Res..

[113] Schwendicke F., Samek W., Krois J. (2020). Artificial Intelligence in Dentistry: Chances and Challenges. J. Dent. Res..

[114] Belstrom D. (2020). The salivary microbiota in health and disease. J. Oral Microbiol..

[115] Kim S., Kim H.J., Song Y., Lee H.A., Kim S., Chung J. (2021). Metabolic phenotyping of saliva to identify possible biomarkers of periodontitis using proton nuclear magnetic resonance. J. Clin. Periodontol..

[116] Ahmad P., Moussa D.G., Siqueira W.L. (2025). Metabolomics for dental caries diagnosis: Past, present, and future. Mass Spectrom. Rev..

[117] Gugnani N., Gugnani S. (2026). AI for caries detection: How close are we to clinical use?. Evid.-Based Dent..

[118] Abbott L.P., Saikia A., Anthonappa R.P. (2025). Artificial Intelligence Platforms in Dental Caries Detection: A Systematic Review and Meta-Analysis. J. Evid.-Based Dent. Pract..

[119] Pornprasertsuk-Damrongsri S., Vachmanus S., Papasratorn D., Kitisubkanchana J., Chaikantha S., Arayasantiparb R., Mongkolwat P. (2025). Clinical application of deep learning for enhanced multistage caries detection in panoramic radiographs. Sci. Rep..

[120] Schwendicke F., Cejudo Grano de Oro J., Garcia Cantu A., Meyer-Lueckel H., Chaurasia A., Krois J. (2022). Artificial Intelligence for Caries Detection: Value of Data and Information. J. Dent. Res..

[121] Asakawa M., Takeshita T., Furuta M., Kageyama S., Takeuchi K., Hata J., Ninomiya T., Yamashita Y. (2018). Tongue Microbiota and Oral Health Status in Community-Dwelling Elderly Adults. mSphere.

[122] Havsed K., Stensson M., Jansson H., Carda-Diéguez M., Pedersen A., Neilands J., Svensäter G., Mira A. (2021). Bacterial Composition and Metabolomics of Dental Plaque From Adolescents. Front. Cell. Infect. Microbiol..

[123] Chen W., Jiang Q., Yan G., Yang D. (2020). The oral microbiome and salivary proteins influence caries in children aged 6 to 8 years. BMC Oral Health.

[124] Gao X., Li Y., Li J., Xiang X., Wu J., Zeng S. (2024). Stimuli-responsive materials in oral diseases: A review. Clin. Oral Investig..

[125] Loya P.R., Nikhade P.P., Paul P., Reche A. (2023). Be Smart and Active in Conservative Dentistry and Endodontics. Cureus.

[126] Abozaid D., Azab A., Bahnsawy M.A., Eldebawy M., Ayad A., Soomro R., Elwakeel E., Mohamed M.A. (2026). Bioactive restorative materials in dentistry: A comprehensive review of mechanisms, clinical applications, and future directions. Odontology.

